# Improving access to direct acting antivirals via a multimodal integrated care program in an addiction medicine clinic

**DOI:** 10.1111/ajad.70155

**Published:** 2026-03-12

**Authors:** Arya Zandvakili, Joseph Rattenni, James Burton, Lakshmi Guduguntla, Marcus Osman, Sayeh Sabbagh, Stephan Arndt, Ben Miskle, Quanhathai Kaewpoowat, Michael Ohl, Andrea Weber, Alison Lynch

**Affiliations:** ^1^ Division of Infectious Diseases University of Iowa Carver College of Medicine Iowa City Iowa USA; ^2^ Department of Family Medicine University of Iowa Carver College of Medicine Iowa City Iowa USA; ^3^ Department of Psychiatry University of Iowa Carver College of Medicine Iowa City Iowa USA; ^4^ Department of Internal Medicine University of Iowa Carver College of Medicine Iowa City Iowa USA; ^5^ Department of Biostatistics University of Iowa College of Public Health Iowa City Iowa USA

## Abstract

**Background and Objectives:**

Injection drug use is a driver of hepatitis C virus (HCV) transmission, thus integrating HCV care into addiction care is likely necessary for HCV elimination. Here we describe how we integrated HCV care into our addiction medicine (AM) clinic and evaluate its effect on HCV treatment.

**Methods:**

Integrated care involves HCV screening and treatment with either DAA prescription directly in AM clinic or via telemedicine with an infectious diseases (ID) specialist. Using retrospective chart review, we assessed if the program affected rates of direct‐acting antivirals (DAA) initiation, DAA completion, and sustained virological response (SVR).

**Results:**

Among 72 treatment naïve patients, the rate of DAA initiation increased after integrated care (HR 2.21, 95% CI 1.05–4.66), but rates of DAA completion or SVR did not significantly increase. Integrated care was associated with more DAA prescriptions (0.6 vs. 0 prescriptions per month, *p* = .004) and decreased referrals to hepatology (0.2 vs. 1 referrals per month, *p* = .001). Compared to referring patients to hepatology, prescribing DAAs in AM clinic was associated with higher rates of DAA initiation (HR 42.46; 95% CI: 15.25–118.24) and completion (HR 8.33; 95% CI: 2.76–25.16).

**Discussion and Conclusions:**

An integrated care program that involved both in‐person and telemedicine options improve access to DAA therapy. Enhancing interprofessional collaboration and expanding telemedicine services offers a practical model for strengthening HCV care delivery.

**Scientific Significance:**

This study demonstrates a practical approach to integrating HCV care into addiction treatment through development of inter‐professional collaboration between healthcare specialties.

## INTRODUCTION

There is a syndemic of substance use disorder (SUD) and hepatitis C, a common bloodborne viral infection that affects approximately 2.5–4.0 million people in the United States (US).^1^ Incidence is highest among adults under 40 years of age and associated with intravenous (IV) substance use.^1^, ^2^ Direct‐acting antivirals (DAAs) are the current standard of care for hepatitis C due to their high efficacy and low rate of adverse reactions. Access to DAAs has been limited by siloing of care and payer restrictions on prescriptions, among other barriers such as stigma and cost of care.^3^

Here, siloing of care refers to treating SUD and hepatitis C in separate settings, with SUD typically treated in addiction medicine (AM) and hepatitis C treated in hepatology or infectious diseases (ID) clinics. Development of simplified hepatitis C treatment protocols^4^ involving DAAs now facilitates treatment in AM and primary care settings, but uptake has been slow.^5^ To counter this, integrated care models have been developed as an effective means for patients with SUD to receive hepatitis C care directly at SUD treatment settings without referral to other specialists. Examples of integrated care include: (1) co‐location of specialists for SUD and hepatitis C within the same clinic^6^; (2) access to hepatitis C treatment via telemedicine at AM clinics^7^; or (3) AM specialists directly prescribing DAAs independently or in consultation with ID specialists.^8^ Integrated care has been associated with improvement in patient satisfaction, appointment attendance, and treatment adherence.^9^, ^10^, ^11^ Despite the empirical evidence supporting integrated care for SUD and hepatitis C, adoption is not widespread.^12^

Due to the high cost of DAAs, insurers in the United States have implemented prior authorization (PA) requirements to limit prescriptions and the financial impact of these medications. For example, some state Medicaid programs, which cover the majority of individuals with hepatitis C,^5^ restricted DAAs to individuals with advanced liver disease and negative drug screens, and also required DAAs to be prescribed by certain specialists.^13^ Such requirements, in particular prescriber restrictions, hamper development of the integrated care models. Recent studies demonstrate that easing of Medicaid PA requirements for DAAs have resulted in increased DAA use,^14^, ^15^ but this effect was not observed in states with low Hepatitis C prevalence^14^—possibly due to underpowering or a true lack of effect.

Iowa is a low‐prevalence state (23.9 confirmed and probable cases of hepatitis C per 100,000 in 2021^16^). Since 2019, Iowa Medicaid has eased prescriber restrictions, liver fibrosis restrictions, and abstinence restrictions on DAAs. This allowed us to establish an integrated care program in our AM clinic in October 2021 involving cooperation of AM specialists, ID specialists, and clinical pharmacists. We show that establishing an integrated care program was associated with increased rates of initiation of DAA therapy, thereby demonstrating a mechanism by which easing Medicaid PA requirements can result in increased access to DAAs, even within a low‐prevalence state.

## METHODS

### Intervention

Previously, patients with hepatitis C at the University of Iowa Health Care (UIHC) AM clinic were referred to hepatology clinic for evaluation and treatment. Iowa Medicaid eased PA requirements for DAA therapy: (1) by covering prescriptions ordered by clinicians who were not ID or gastroenterology specialists (but “in consultation with a digestive disease, liver disease, or infectious disease provider practice”) in January 2019^17^; and (2) removed requirements for advanced liver disease in July 2020.^18^ Additionally, Iowa Medicaid removed the PA requirement for a specific period of abstinence from substance use prior to initiation of DAA therapy in October 2022.^19^

The above changes motivated AM and ID specialists to develop an integrated care program for hepatitis C in AM clinic. The development of the program involved input from AM specialists, ID specialists, clinical pharmacists, case managers, AM clinic nurses, and AM clinic schedulers. Initial meetings involved process mapping to determine feasibility of the project and identify a workflow to implement the integrated care project.

A process was developed to identify patients eligible for a simplified hepatitis C treatment protocol.^4^ Criteria for eligibility were that patients must be nonpregnant, hepatitis B negative, HIV negative, hepatitis C treatment naïve, and low risk of cirrhosis (based on FIB‐4^20^ index <1.45 without other evidence of cirrhosis). Patients with an intermediate FIB‐4 index (i.e., 1.45–3.25) were evaluated for risk of cirrhosis based on additional clinical data including history, physical exam, and prior imaging results. An order set within the Epic electronic health record was created that included necessary lab tests for assessing eligibility. If needed, case managers within the clinic would gather laboratory results from external sources to facilitate eligibility determination. Patients not meeting criteria for the simplified treatment protocol were offered referral to a hepatology clinic through the historical pathway.

Medicaid policy still required consultation with specialists prior to DAA prescription. Therefore, an e‐consultation process was developed whereby AM specialists would include in their clinic note a plan for hepatitis C treatment and co‐sign their electronic note to an ID specialist. The ID specialists would review that chart and provide written feedback if needed.

Transportation barriers may prevent patients from coming to UIHC campus for in‐person visits to be seen or obtain labs. Therefore, a telemedicine component was incorporated into the program. This telemedicine component was staffed by an ID specialist.

The integrated care program started in October 2021. A subset of clinicians within the AM clinic reviewed their patients' hepatitis C history and performed hepatitis C virus (HCV) serum antibody screening or quantitative serum HCV RNA testing as needed. Patients who meet simplified treatment criteria were offered DAA therapy in‐person at AM clinic (with e‐consultation with an ID specialist) or offered hepatitis C care via telemedicine based on patient or clinician preference. Clinical pharmacists embedded in the AM clinic had a collaborative practice agreement for hepatitis C testing and treatment. These pharmacists could order laboratory tests and prescribe DAA therapy in consultation with the AM and ID specialist. These pharmacists also provided education and monitor adherence and tolerance to DAA therapy through telephone calls with patients.

### Study design

We conducted a retrospective cohort study of patients with hepatitis C who were seen at the UIHC AM clinic between 01/01/2019 and 05/31/2023. We compared rates of DAA initiation, DAA completion, and SVR before and after implementation of the integrated care program. We also compared rates of hepatitis C treatment depending on the hepatitis C intervention employed by AM clinic: prescribe DAA via integrated care, refer to hepatology, or other/no intervention. This does not account for interventions employed by other clinic services. This research was reviewed and approved by the UIHC's Institutional Review Board.

### Cohort

We queried UIHC's electronic medical record using Epic's “Slicer‐Dicer” tool and found that 1088 patients had scheduled appointments at the AM clinic during the study period (01/01/2019–05/31/2023). Of those, 146 had a history of a positive serum HCV antibody, positive serum HCV RNA, or an ICD‐10 diagnosis for hepatitis C (B17.10, B17.11, B18.2, B19.20, B19.21, R76.8, Z86.19). Using manual chart review (see Chart Review & Data Collection below), 72 patients were identified as having untreated hepatitis C while receiving care from AM clinic during the study period (Figure S1).

### Chart review and data collection

We used manual chart review of the electronic medical record (including outside records available via Epic's “Care Everywhere” function and scanned documents) to collect data on HCV RNA level history, hepatology referrals for hepatitis C, DAA therapy history (prescriber, DAA prescribed, date of prescription, data of DAA initiation, date of DAA completion, adverse effects, and disruptions in therapy), liver function test results, medical and psychiatric comorbidities, substance use history, and death. Six of the authors were involved in chart review (JB, LG, MO, JR, SS, AZ). Each patient in the cohort was reviewed by at least two reviewers, and any discrepancy between the reviewers resulted in additional review by either a third reviewer or one of the two original reviewers to resolve discrepancies. Additional data on patient demographics (date of birth, legal sex, race, ethnicity, and county of residence), AM clinic visit dates, and AM clinic lab orders were collected directly from Epic. Data from chart review was entered into Research Electronic Data Capture (REDCap).^21^

### Outcomes

Primary outcome measures were time‐to‐DAA initiation, time‐to‐DAA completion, and time‐to‐SVR. Time‐to‐event was measured in reference to the first AM clinic appointment after a patient is known to have active hepatitis C based on chart review or the date of the first positive HCV RNA level if ordered by AM clinician, whichever was earlier. If this date was prior to 01/01/2019, the time started on 01/01/2019. Patients were censored if they died, were lost to follow‐up (determined as no AM clinic appointment for >1 year), or no event occurred by the end of study period (08/31/2023).

Process measures were number of referrals to hepatology per month and number of DAA prescriptions per month.

### Data analysis and statistics

An unadjusted Cox regression model was used to compare outcome measures by program status (i.e., before vs. after starting the integrated care program). The program status was treated as a time‐dependent variable to account for patients that were present both before and after starting integrated care.

An adjusted Cox regression model was also used to compare outcomes based on intervention employed by AM clinic among patients seen after Medicaid policy was relaxed (January 2019): prescribe DAA via integrated care, refer to hepatology, or neither (“no/other intervention”). The intervention variable was treated as a time‐dependent variable that was updated at each AM clinic visit. Once the variable is “prescribed DAA” or “referred to hepatology,” it was not updated to “other/no intervention” until at least 1 year had passed without an outcome of interest occurring.

Trends in process measures were measured using an unadjusted binomial regression model. Outcomes based on which clinic prescribed DAA were compared using Fischer exact test.

All data was processed and analyzed using R. Scripts used for data processing, statistics, and generating plots are available at github.com/aryazand/hepc_integrated_care.

## RESULTS

### Patient characteristics

Between January 1, 2019 and May 31, 2023, 72 patients with treatment‐naïve hepatitis C were seen at AM clinic (Figure S1). Fifty of the patients were seen in AM clinic before integrated care (January 2019 to September 2021) and 57 of the patients were seen in AM clinic after starting integrated care (October 2021 to August 2023) (Table 1). Of note, 35 patients were observed both before and after starting integrated care. Two‐thirds of the patients were male, and nearly all were in the 4th decade of life. Only three patients had advanced liver fibrosis predicted by a FIB‐4 > 3.25. More than half the patients had depression and anxiety (57% and 58%, respectively), and approximately one third had posttraumatic stress disorder (PTSD) (31%). Nearly all patients had a history of opioid use disorder (97%) and a history IV drug use (94%).

**Table 1:** Patient demographics, comorbidities, and substance‐use history.

	All	Before starting integrated care	After starting integrated care
Characteristic	*N* = 72^a^	*N* = 50^a^	*N* = 57^a^
Demographics			
Sex			
Female	23 (32%)	17 (34%)	20 (35%)
Age	34 (31, 38)	34 (30, 38)	35 (32, 41)
Race			
White	61 (85%)	42 (84%)	48 (84%)
Latino/a of any race	4 (5.6%)	3 (6.0%)	4 (7.0%)
Black	4 (5.6%)	3 (6.0%)	4 (7.0%)
Other	3 (4.2%)	2 (4.0%)	1 (1.8%)
Ethnicity			
Non‐Hispanic	66 (92%)	46 (92%)	51 (89%)
Hispanic	4 (5.6%)	3 (6.0%)	4 (7.0%)
Unknown	2 (2.8%)	1 (2.0%)	2 (3.5%)
Medical comorbidities			
Diabetes	1 (1.4%)	1 (2.0%)	1 (1.8%)
HIV	0 (0%)	0 (0%)	0 (0%)
ESRD	1 (1.4%)	1 (2.0%)	1 (1.8%)
Cirrhosis	2 (2.8%)	1 (2.0%)	2 (3.5%)
Prior hepatitis C therapy	0 (0%)	0 (0%)	0 (0%)
FIB‐4	0.8 (0.6, 1.1)	0.8 (0.7, 1.2)	0.8 (0.6, 1.1)
FIB‐4 > 3.25	3 (5.1)	2 (4.7)	3 (6.7)
Unknown	13	7	12
Psychiatric comorbidities			
Depression	41 (57%)	28 (56%)	31 (54%)
Anxiety/Panic	42 (58%)	31 (62%)	32 (56%)
PTSD	22 (31%)	17 (34%)	15 (26%)
Bipolar	3 (4.2%)	2 (4.0%)	3 (5.3%)
Schizophrenia	1 (1.4%)	0 (0%)	1 (1.8%)
Other Psychiatric History	22 (31%)	13 (26%)	17 (30%)
Substance Use Disorder History			
Opioid	70 (97%)	49 (98%)	55 (96%)
Alcohol	17 (24%)	12 (24%)	13 (23%)
Methamphetamine	40 (56%)	28 (56%)	30 (53%)
Tobacco	20 (28%)	15 (30%)	16 (28%)
Other Substance	17 (24%)	12 (24%)	11 (19%)
IV Drug Use History	68 (94%)	47 (94%)	53 (93%)

*Note*: Values are separated by patients who were present in cohort before and after starting integrated care. Some patients (*N* = 35) were observed in both periods.

^a^ *n* (%); Median (Q1, Q3).

### Primary outcomes

We modeled the hepatitis C treatment as a cascade of four stages: DAA prescription, DAA initiation, DAA completion, and SVR—with the last three stages being primary outcomes.

**Figure 1:**
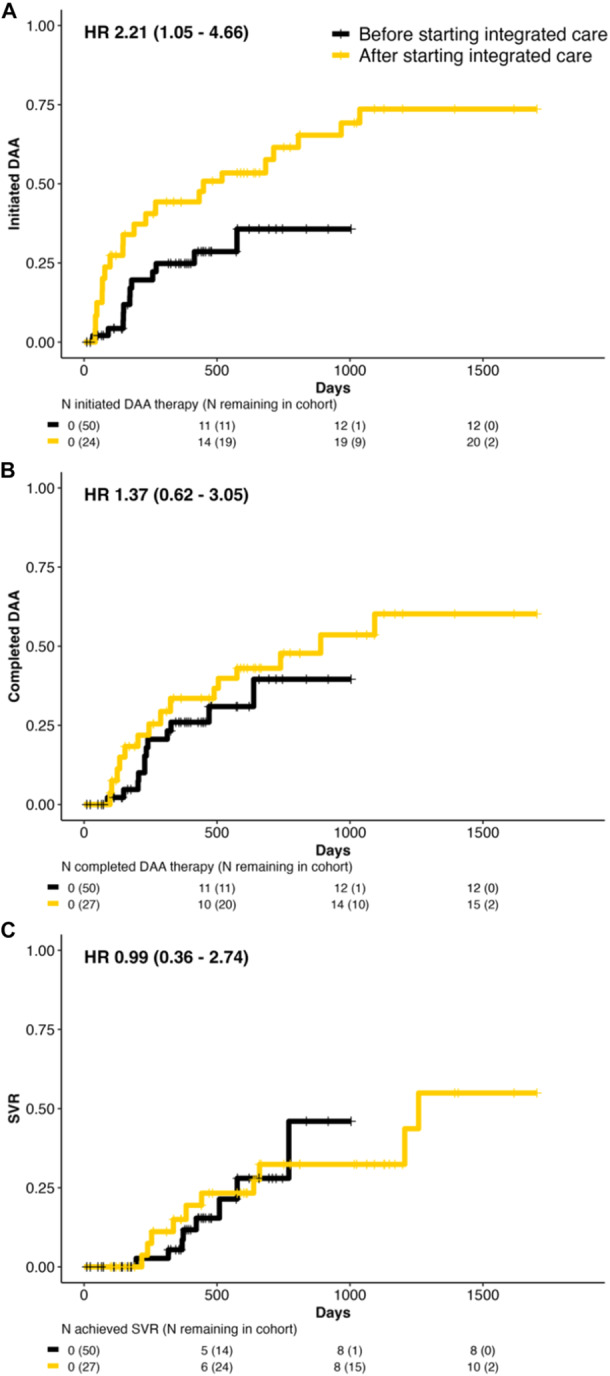
Rates of DAA initiation, DAA completion, and SVR before versus after starting integrated care. Survival curves demonstrating time‐to‐initiation of DAA therapy (A), time‐to‐initiation of DAA therapy (B), and time‐to‐SVR (C). Vertical lines represent censoring. Hazard‐ratios calculated by unadjusted Cox‐proportional hazards models. DAA, Direct Acting Antiviral; HR, hazard ratio.

After starting the integrated care program, the rate of DAA initiation significantly increased (42.8 vs. 24.5 per 100 patient‐years; HR 2.21, 95% CI 1.05–4.66) (Figure 1A). However, no significant change was detected in the rate of DAA completion (29.1 vs. 23.7 per 100 patient‐years; HR 1.37, 95% CI 0.62–3.05) or SVR (15.6 vs. 14.4 per 100 patient‐years; HR 0.36–2.74) (Figure 1B,C).

There was an increase in the raw proportion of patients initiating DAA therapy (44% vs. 26%) after versus before integrated care (23 vs. 33 months) (Figure S2). A similar proportion of patients had completed DAA therapy (28% vs. 24%) and were confirmed SVR (18% vs. 16%) after versus before starting integrated care, despite the observation period after starting integrated care being substantially shorter than before starting integrated care. Most treatment gains occurred among patients who had not been prescribed or initiated DAA therapy prior to integrated care. There were only two patients who began the treatment cascade before the integrated care program who then advanced along the treatment cascade after the start of integrated care (Figure S2).

### Process measures

After starting integrated care there was a significant reduction in hepatology referrals (0.2 vs. 1 referrals per month, *p* = .004) and a significant increase in DAA prescriptions from AM clinic (0.6 vs. 0 DAA prescriptions per month, *p* = .001) for the patients in the cohort (Figure S3). There was not a significant change in the number of DAA prescriptions from hepatology clinic for the patients in the cohort compared to before integrated care (0.5 vs. 0.6 DAA prescriptions per month, *p* = .514).

**Figure 2:**
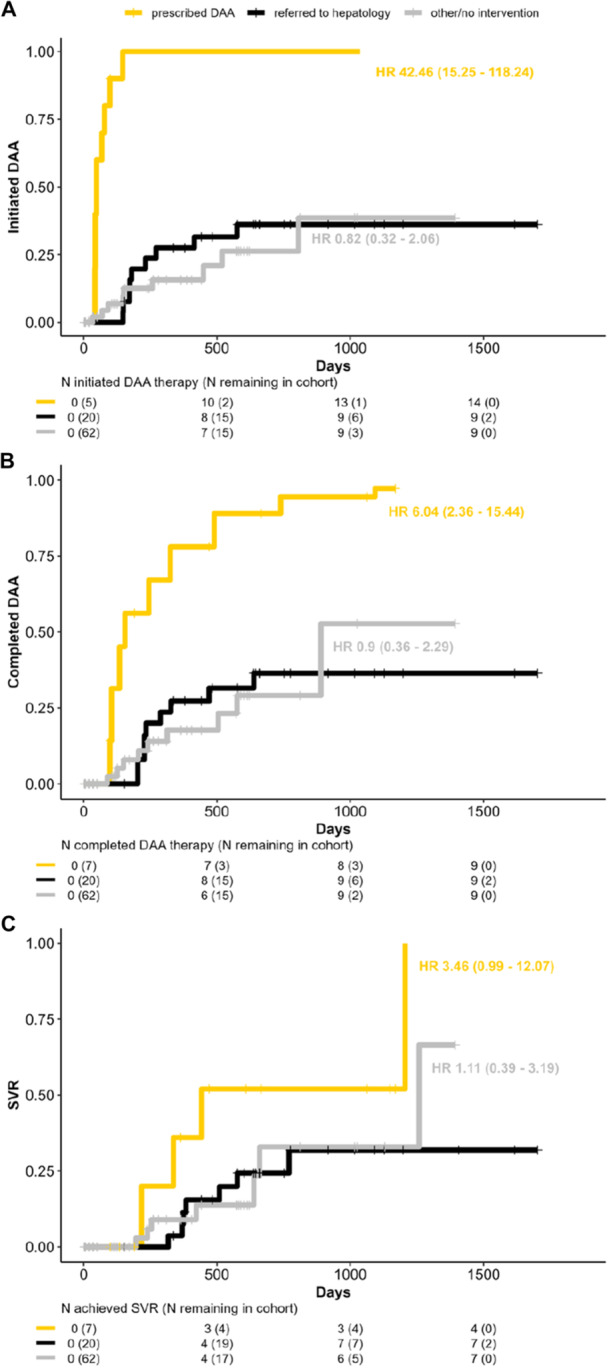
Time‐to‐treatment of hepatitis C based on management strategy employed in integrated care. After implementing integrated care, clinicians in AM clinic could have managed hepatitis C via three strategies: “prescribed DAA,” “referred to hepatology,” “other/no intervention.” Survival curves demonstrating time‐to‐initiation of DAA therapy (A), time‐to‐initiation of DAA therapy (B), and time‐to‐SVR (C) based on the management strategy. Vertical lines represent censoring. Hazard ratios calculated by proportional hazards model with hepatitis C management strategy as a time‐varying variable. DAA, Direct Acting Antiviral; HR, Hazard Ratio.

To assess if the increased rate of DAA initiation after starting the integrated care program was specifically associated with DAA prescriptions from AM clinic, we compared outcomes for patients receiving prescriptions via integrated care program versus referrals to hepatology clinic after the integrated care program started. We limited our analysis to the period after Medicaid restrictions on DAA therapy began to relax (starting January 2019). Indeed, there was an increased rate of DAA initiation (777.0 vs. 19.9 per 100 patient‐years; HR 42.46; 95% CI: 15.25–118.24) and DAA completion (118.0 vs. 19.3 per 100 patient‐years; HR 8.33; 95% CI: 2.76–25.16) when receiving a DAA prescription from integrated care compared receiving a referral to hepatology. There was not a statistically significant increase in the rate of SVR (36.6 vs. 13.1 per 100 patient‐years; HR 3.24; 95% CI: 0.80–13.11) (Figure 2). When AM clinic referred to hepatology there was no difference in rates of DAA initiation, DAA completion, or SVR compared to when AM clinic performed other/no intervention.

Overall, 15 patients received DAA prescriptions from hepatology clinic, 16 patients received DAA prescriptions from AM clinic, and one patient received treatment from a clinician outside our healthcare system (Table 2). All DAA prescriptions from integrated care were for glecaprevir/pibrentasvir, whereas 11 out of 15 DAA prescriptions from hepatology clinic were for glecaprevir/pibrentasvir (the rest were for sofosbuvir‐velpatasvir). Regardless of prescribing clinic, a similar proportion of patients started and completed medications. Only 40% of patients who received DAA prescription from integrated care were tested for SVR, compared to 75% of patients who received DAA prescription from hepatology were tested for SVR. Regardless of prescribing clinic, 100% of patients who were tested for SVR had achieved SVR.

**Table 2:** Antiviral therapy outcomes by prescribing clinic.

	Prescribing clinic			
	Hepatology *N* = 15^a^	Integrated Care *N* = 16^a^	Other *N* = 1^a^	*p*‐value^b^
DAA therapy				.066
Glecaprevir‐pibrentasvir	11 (73%)	16 (100%)	1 (100%)	
Sofosbuvir‐valpastavir	4 (27%)	0 (0%)	0 (0%)	
Prior authorization denied	3 (20%)	1 (6.3%)	0 (0%)	.4
DAA initiated	13 (87%)	15 (94%)	1 (100%)	.6
DAA completed				.3
Yes	12 (80%)	10 (63%)	1 (100%)	
No	3 (20%)	2 (13%)	0 (0%)	
Unknown	0 (0%)	4 (25%)	0 (0%)	
Checked for SVR	10 (67%)	4 (25%)	1 (100%)	.021
SVR	10 (67%)	4 (25%)	1 (100%)	.021

*Note*: Percentages are of total patients in each column.

Abbreviations: DAA, direct acting antiviral; SVR, sustained virological response.

^a^ *n* (%).

^b^ Fisher's exact test.

The integrated care program allowed patients to receive hepatitis C care in clinic from an AM specialist or via telemedicine from an ID specialist based on patient and clinician preference. Out of the 16 DAA prescriptions that were prescribed from the integrated care program during the study period, 5 (31%) were prescribed via telemedicine by an ID specialist.

## DISCUSSION

This study demonstrates how the implementation of an integrated care program, made possible by easing of Medicaid requirements for DAA prescriptions, was associated with improvements in access to DAA therapy. The integrated care program involved AM specialists providing direct patient care, ID specialists providing e‐consults and telemedicine appointments, and clinical pharmacists assessing safety, tolerance, and adherence of medications. The program streamlined hepatitis C treatment for individuals with substance use disorders, a population that has historically faced numerous barriers to accessing timely and effective care and is underserved by conventional healthcare systems. The findings highlight the potential of such integrated care models to enhance hepatitis C treatment outcomes and reduce the public health burden of hepatitis C, particularly in the context of policy changes that facilitate broader access to antiviral therapy.

### Integrated care was associated with an improvement in DAA access

After implementing the integrated care program there was a statistically significant increase in the rate of DAA initiation but not rates of DAA completion or SVR (Figure 1). To assess if this effect was a specifically associated with integrated care, we compared primary outcomes of patients who received DAA prescriptions versus GI referrals from AM clinic for Hep C treatment. Indeed, prescribing DAA therapy in the AM clinic was associated with a statistically significant increased rate of DAA completion compared to referring patients to hepatology (Figure 2). Overall, our results are consistent with other studies that have shown integrated care improves treatment outcomes for hepatitis C among people with SUD.^6^, ^7^, ^8^, ^10^, ^22^

### The role of telemedicine

The integrated care program was multimodal, in that it allowed patients to receive hepatitis C care directly in clinic from an AM specialist or via telemedicine from an ID specialist. The telemedicine option provided flexibility based on preferences of the patient (e.g., a patient who preferred not to travel to clinic for hepatitis C care) and the AM clinician (e.g., an AM clinician who does not feel comfortable treating hepatitis C). Approximately one third of the DAA prescription from integrated care occurred via telemedicine.

### The role of e‐consultation with ID

At the time of this study, Iowa Medicaid required DAAs to be prescribed “in consultation with a digestive disease, liver disease, or infectious disease” specialist. Thus, our integrated care program implemented an e‐consultation process with ID specialists. The e‐consultations provided useful guidance to AM specialist in treating hepatitis C, particularly in cases that were complex. However, it was also an additional administrative burden in cases that were deemed straightforward. Notably, Iowa Medicaid has removed the requirement for e‐consultation after the study period ended. Further study will be needed to assess how the removal of this requirement affected the integrated care program.

### Impact on medicaid policy on implementation of integrated care

Importantly, implementation of our integrated care within the AM clinic was not feasible under restrictive Medicaid policy that limited DAA prescribing only to ID, gastroenterology, and hepatology specialists. By easing restrictions, AM specialists were able to directly prescribe DAAs, which allowed for program implementation. A recent study found that easing DAA restrictions by Medicaid between 2015 and 2019, resulted in increased rates of HCV treatment in high and medium HCV prevalence states, but this effect was not observed in low prevalence states.^14^ In contrast, we found that easing Medicaid restrictions in Iowa (a state with low HCV prevalence) was associated with increased DAA access among our patient population. This difference in results may be because the initial study did not have statistical power to detect effects in lower prevalence states or because the initial study occurred prior to Iowa's policy changes (thus Iowa wasn't included in the study). Our results suggest that easing Medicaid restrictions on its own may not be sufficient to increase hepatitis C treatment but may facilitate novel programs that are necessary to improve rates of hepatitis C treatment.

### Generalizability of the integrated care program

Our AM clinic is housed within the Psychiatry Department of a tertiary care center, which was conducive to developing inter‐professional collaboration (between psychiatrists, ID specialists, pharmacists, and nurses) needed for the integrated care program. To implement a similar program at standalone addiction clinics may require developing relationships with pharmacists and ID specialists at local clinics or hospitals.

### Strengths and limitations of this study

The strengths of this project include the implementation of a novel interdisciplinary care model, the use of high‐quality, real‐world data from a single‐center cohort, and the evaluation of policy changes on treatment outcomes.

Our study has multiple limitations. First, the generalizability of our intervention and results are limited by small sample size and relatively homogenous patient demographics (the patients in our cohort were predominately white, male, and under 40 years of age). Factors contributing to this limitation include epidemiological factors (i.e., relative low prevalence of hepatitis C in Iowa and racial demographics of hepatitis C in Iowa^23^), issues with access, psychosocial factors (e.g., stigma), healthcare‐related factors (e.g., during the study Iowa Medicaid necessitated consult with an ID or gastroenterologist to approve DAA therapy), and study design factors (e.g., single‐center study).

Second, our result that AM clinic directly prescribing DAAs resulted in a higher rate of DAA completion likely suffers from selection bias, as complex liver patients were referred to hepatology, and their liver disease may confound DAA completion rates.

Third, there was a low rate of testing for SVR among the patients receiving hepatitis C treatment from integrated care (4 out of 10 patients who completed DAA therapy, Table 2). Contributing factors may include clinicians not prioritizing testing for SVR compared to other needs of the patient, patient‐specific barriers creating challenges in returning for phlebotomy, the complexities of ordering and completing labs at external sites (for patients living far from AM clinic or when AM visits were conducted via telemedicine). Study design factors also contribute, namely that patients who received DAA prescription from integrated care tended to receive the prescription later in the study and thus had less opportunity tested for SVR. The low rate of testing for SVR likely contributed to the lack of significant change in SVR rate after starting integrated care. Strategies to improve rates of SVR testing should be considered for future improvement and implementation of integrated care programs.

## CONCLUSIONS

This study highlights integrated care programs as a potential mechanism for improving hepatitis C treatment among underserved populations when government policy expands access to hepatitis C therapies. Further studies are needed to assess how to optimize and expand integrated care programs to achieve hepatitis C eradication.

## CONFLICT OF INTEREST STATEMENT

The authors declare no conflicts of interest. The authors alone are responsible for the content and writing of this paper.

## Supporting information

Figure S1.

Figure S2.

Figure S3.
