# Supplementary material for: Improving access to direct acting antivirals via a multimodal integrated care program in an addiction medicine clinic
Source: Am J Addict. 2026 Mar 12;35(4):534–42. doi: 10.1111/ajad.70155 (PMC13272766; doi:10.1111/ajad.70155)
Supplement: Supplementary file 1 — Figure S1. [file AJAD-35-534-s003.pdf]

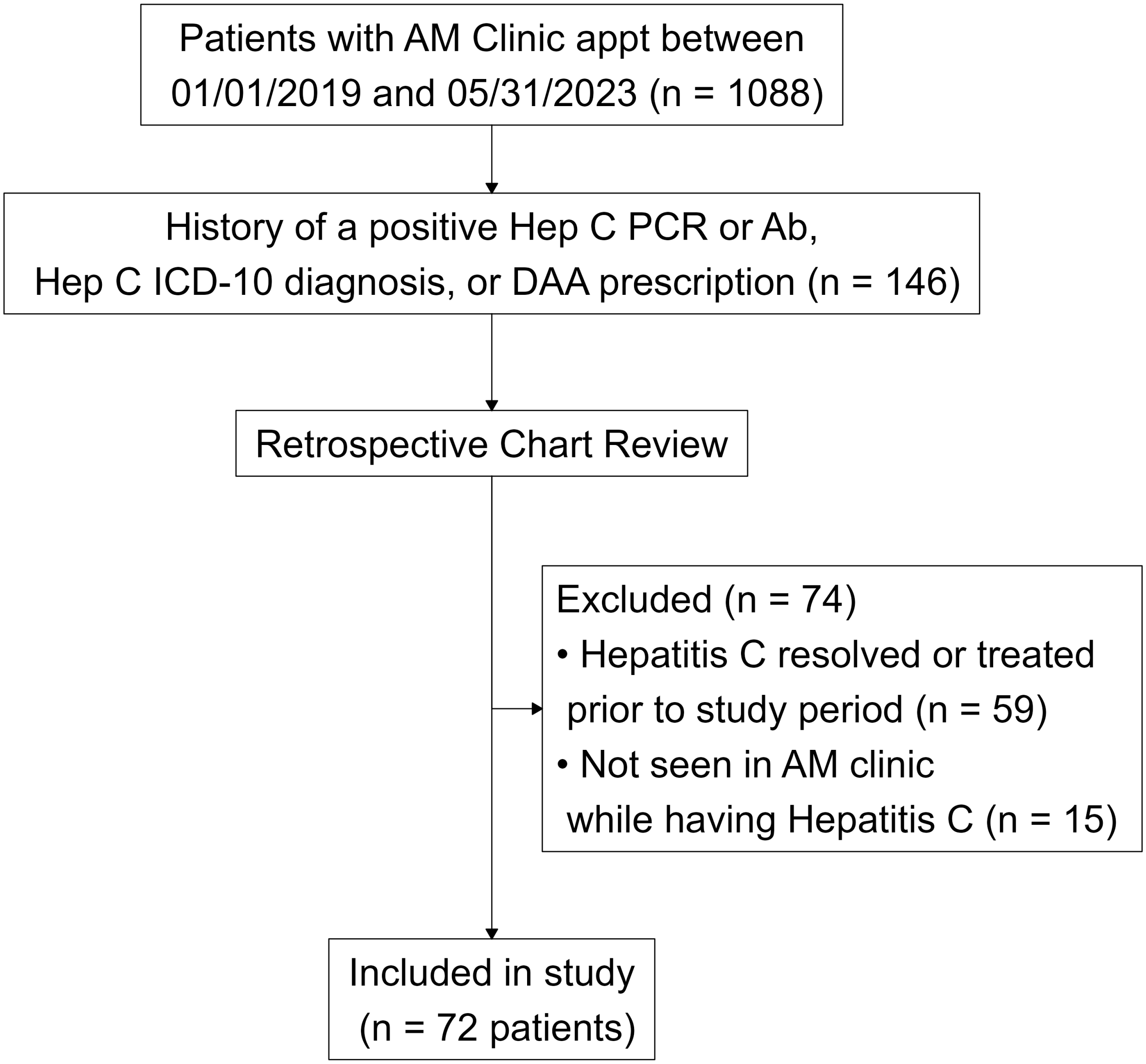

Figure S1. Consort diagram demonstrating the selection of Addiction Medicine (AM) clinic patients with hepatitis C included in study. Ab = “Antibody”; DAA = “Direct Acting Antiviral”; PCR = “Polymerase Chain Reaction”.
