# Supplementary material for: Improving access to direct acting antivirals via a multimodal integrated care program in an addiction medicine clinic
Source: Am J Addict. 2026 Mar 12;35(4):534–42. doi: 10.1111/ajad.70155 (PMC13272766; doi:10.1111/ajad.70155)
Supplement: Supplementary file 2 — Figure S2. [file AJAD-35-534-s001.pdf]

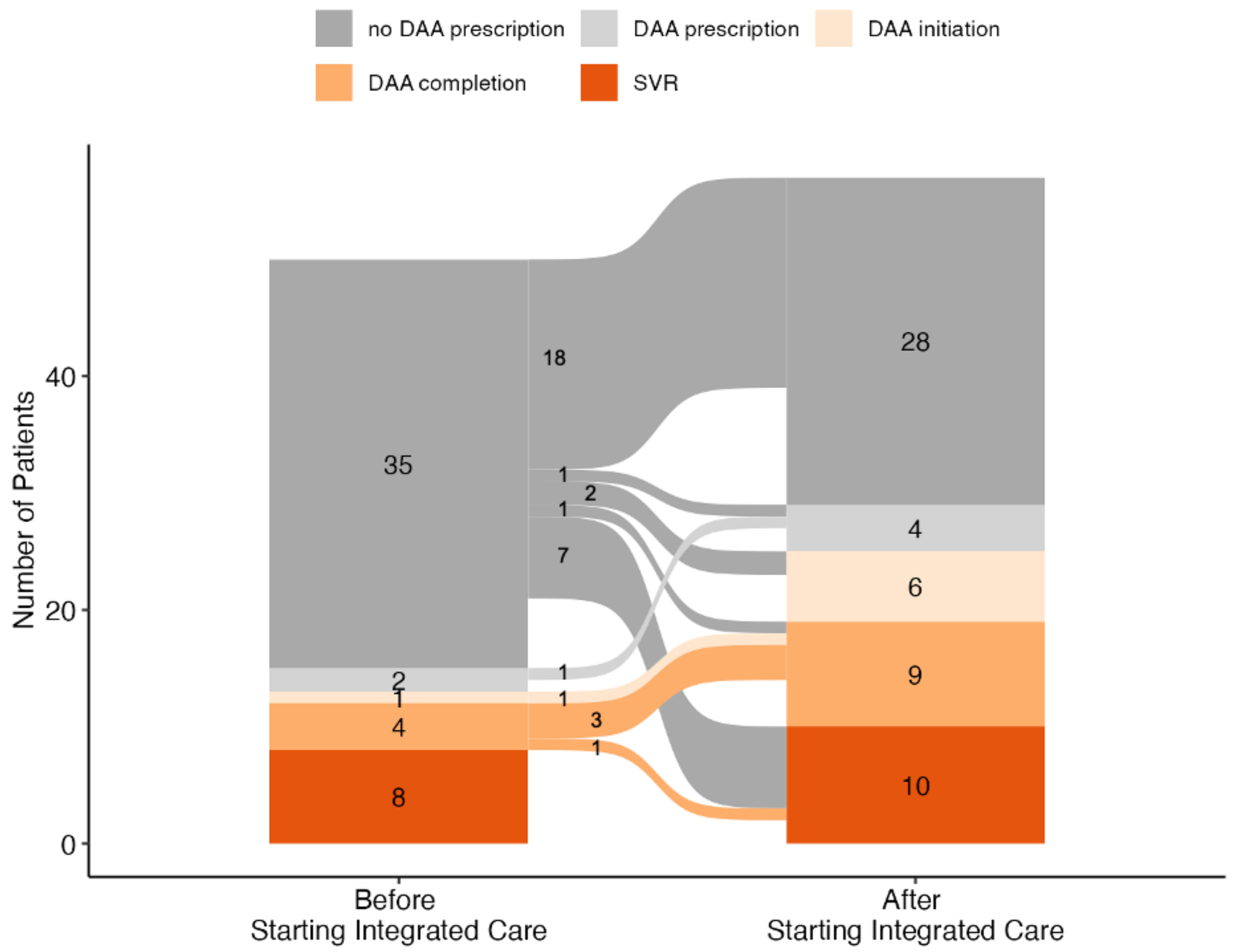


**Figure S2. Number of patients in each stage of hepatitis C treatment cascade before and after starting integrated care program.** Distribution of patients among different hepatitis C treatment states (untreated, prescribed DAA, initiated DAA, completed DAA, or SVR) before (33 months) and after (23 months) starting the integrated care. Ribbons with numbers between stacks show how many patients progressed in treatment states between the two observation periods.
