# Supplementary material for: Improving access to direct acting antivirals via a multimodal integrated care program in an addiction medicine clinic
Source: Am J Addict. 2026 Mar 12;35(4):534–42. doi: 10.1111/ajad.70155 (PMC13272766; doi:10.1111/ajad.70155)
Supplement: Supplementary file 3 — Figure S3. [file AJAD-35-534-s002.pdf]

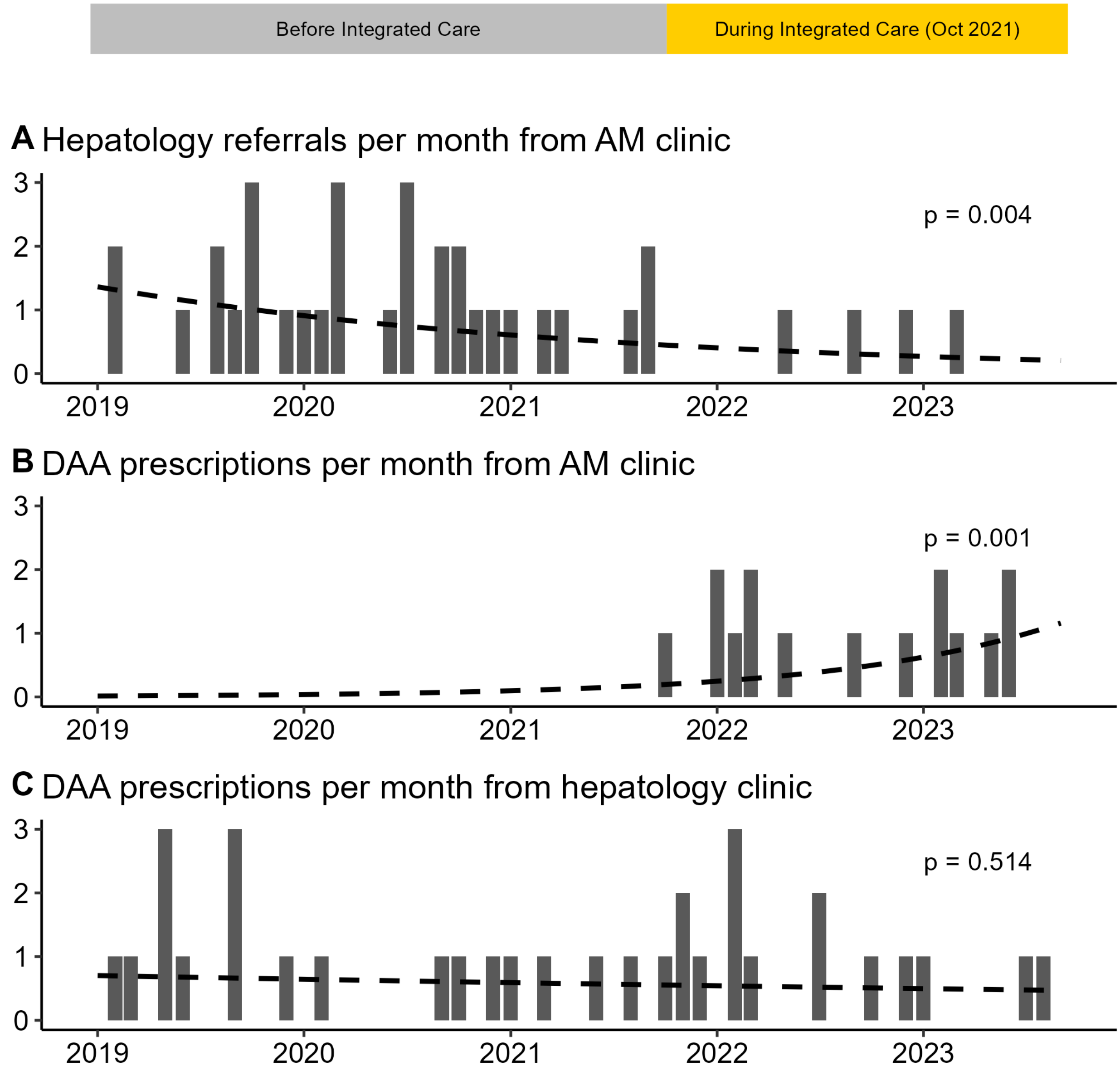

Figure S3. Change in hepatology referrals and DAA prescriptions after easing of Medicaid restrictions and implementation of integrated care. *Top panel*: Timeline demonstrating when integrated care was implemented. **(A)** Hepatology referral per month from AM clinic. **(B)** DAA prescription per month from AM clinic. **(C)** DAA prescriptions per month from hepatology clinic. Dotted curves represent negative binomial regressions to measure change in referrals and DAA prescriptions over time. AM = “Addiction Medicine”; DAA = “Direct Acting Antiviral”.
